# Professionals’ perceptions about healthcare resources for co-occuring disorders in Spain

**DOI:** 10.1186/1752-4458-8-35

**Published:** 2014-08-28

**Authors:** Carlos Roncero, Pablo Vega, Jose Martínez-Raga, Carmen Barral, Ignacio Basurte-Villamor, Laia Rodríguez-Cintas, Beatriz Mesías, Lara Grau-López, Miguel Casas, Nestor Szerman

**Affiliations:** Sociedad Española de Patología Dual. Londres, 17 28028 Madrid, EU Spain; Department of Psychiatry, Outpatient Drug Clinic, Vall d’Hebron University Hospital - Public Health Agency, Barcelona (ASPB), CIBERSAM. Passeig de la Vall d’Hebrón, 119-129, 08035 Barcelona, EU Spain; Department of Psychiatry and Legal Medicine, Universitat Autònoma de Barcelona, Plaça Cívica, 08193 Bellaterra (Cerdanyola del Vallès), Barcelona, EU Spain; Instituto de Adicciones, C/ Juan Esplandiú, 11-13, 28007 Madrid, EU Spain; Unidad Docente de Psiquiatría y Psicología Medica, Hospital Universitario Dr. Peset y Universidad de Valencia, & Universidad CEU-UCH, 46017 Valencia, EU Spain; Department of Psychiatry, Hospital Universitario Gregorio Marañón, Calle Doctor Esquerdo, 46, 28007, EU Madrid, Spain

**Keywords:** Dual pathology, Dual disorders, Co-occurring disorders, Resources, Professionals’ perception, Mental health, Drug abuse

## Abstract

**Background:**

Since provision of integrated services for patients with dual pathology or dual disorders (coexistence of an addictive disorder and other psychiatric disorders) constitutes an important challenge, this study compared the perceptions of health-care professionals with the existing, current state of specific resources for patients with dual pathology in Spain.

**Methods:**

Epidemiological, observational, cross-sectional, multicenter study with a large, representative sample of health care professionals attending patients with dual pathology in treatment resources throughout Spain. Participants completed a specifically designed *ad*-*hoc* on-line questionnaire about their perceptions on the existence of available resources and treatment needs for patients with dual pathology. To compare professionals’ perceptions with existing available resources, the same on-line questionnaire was also completed by commissioners and managers responsible for national and regional healthcare plans on drug abuse.

**Results:**

A total of 659 professionals, mostly psychologists (43.40%) or psychiatrists (32.93%) agreed to participate in the study. The highest degree of concordance between the perceptions of professional and the actual situation was found regarding the existence of mental health and addiction networks (either separately or unified) (74.48%), followed by specific workshops (73.08%) and sub-acute inpatient units (67.38%), specific hospitalization units (66.26%), detoxification units (63.15%) and outpatient programs (60.73%). We detected a lower degree of agreement regarding specific occupational rehabilitation centers (59.34%) day hospitals (58.93%), day centers (57.88%), outpatient intermediate resources (48.87%), psychiatric acute admission units (46.54%) and therapeutic communities (43.77%). In addition, on average, health care professionals underestimated the number of resources present in their respective communities.

**Conclusions:**

Relevant differences exist between the perceptions of professional and existing available resources for dual pathology patients in Spain, thus supporting the need of additional efforts and strategies to establish a registry and clearly inform about available resources for patients with dual diagnosis.

## Background

Dual pathology, dual disorders or co-occurring disorders are defined as the presence of an addictive and another mental disorder, with rates well-documented above 50% [1–14]. Dually-diagnosed patients represent a substantial proportion of individuals in treatment and commonly show greater severity from both the clinical and social perspectives than individuals with solely one of the disorders [15]. Importantly, dual disorders are usually associated with a significantly worse clinical course and outcome, as well as with a poorer treatment response and adherence, than patients with one of the disorders alone [15–17], as well as with frequent, high levels of polymedication [18]. Failure to detect, diagnose and adequately treat patients with dual pathology can jeopardize their chances of success [19].

Implementation of specific services and resources for dual pathology represents an important challenge [13, 19]. Traditionally, mental health and addiction treatment services have worked sequentially or in parallel, resulting in low adherence of dually diagnosed patients to treatment and in an inadequate management of the common interrelated problems of the comorbidity [17, 20, 21].

Specific integrated resources are being currently developed for patients with dual pathology (such as in- and outpatient units, or day centers). The integrated model envisages a global treatment plan for both mental health disorders and substance abuse disorders, being provided in one time by a multidisciplinary treatment team. Shared treatment plans, as "integrated model" implicates, would minimize philosophical differences among care providers. Substance abuse and psychiatric illnesses are accurately diagnosed and targeted for a stage-specific treatment [15]. In Spain, special resources have developed (including acute inpatient dual diagnosis units; dual diagnosis residential communities; dual diagnosis programs in both mental health and drug user treatment outpatients centers) to move into a final integrated model of treatment [15].

However, to date few studies, focused in professionals’ evaluation about co-occurring resources, have assessed the implementation of these strategies for dually diagnosed patients [22–25]. In a recent epidemiological, observational, cross-sectional, multicenter study conducted in Spain with a sample of healthcare professionals managing patients with co-occurring disorders, according to the perceptions of professionals, specific healthcare resources for co-occurring disorders were clearly insufficient, with scarcity of specific outpatient programs, hospitalization units, sub-acute inpatient units, outpatient intermediate resources, day hospitals and day centers [24]. In fact, the importance of the professionals’ perceptions on other aspects of the disorder (such as management and long-term care) has also been recently addressed [26]. Based on these results, the need of additional efforts and strategies for treating individuals with co-morbid disorders, such as a National Plan on Drugs on co-occurring disorders was suggested [24].

The present study was designed to compare the knowledge of professionals on specific resources for co-occurring disorders.

## Methods

### Design

This observational, cross-sectional, multicenter study was conducted in Spain between February and May 2011 with a representative sample of 659 healthcare professionals involved in the management of patients with co-occurring disorders in various treatment settings throughout Spain, in order to explore the available healthcare resources and the specific needs for patients with co-occurring disorders.

The study protocol was approved by the Ethics Committee of Hospital de la Vall d’Hebrón (Barcelona, Spain) and procedures were in accordance with the ethical standards of the Helsinki Declaration, as revised in 2000. After complete description of the study, healthcare professionals accepted to voluntarily participate, without receiving any remuneration.

### Questionnaire

For this purpose, an *ad*-*hoc* on-line questionnaire ( http://www.patologiadual.es/profesional_publica.html) was designed by a group of different experts in dual diagnosis from different clinical and academic origins, including the authors of this article, and distributed on-line to the participating investigators to collect all relevant data related to specific healthcare resources for patients with co-occurring disorders (available on http://www.sepd.es). In addition, the questionnaire included several items to address the perceptions of the professionals on current available resources for co-occurring disorders (such as specific outpatient units and programs, hospitalization units, detoxification units, day centers, acute admission units, occupational rehabilitation programs and centers, or therapeutical communities) and resources that, in their opinion, should be made available for patients with co-occurring disorders or dual disorders. Their opinion on the integration model was also solicited. The results of this on-line questionnaire has been reported previously [24].

To compare professionals’ perceptions with existing available resources, the same on-line questionnaire was also completed by commissioners and managers responsible for national and regional healthcare plans on drug abuse (between June and July 2012). The degree of agreement or concordance between professionals’ perceptions and commissioners was expressed as percentages of surveyed healthcare professionals informed on the number of different resources for dual pathology.

All the members of the Spanish Society of Dual pathology (SEPD) and the professionals in the SEPD database (over 2,000) were sent three e-mails inviting them to complete the questionnaire. The questionnaire was also available to all the professionals working in the mental health or addiction field in Spain who accessed the SEPD website.

We assessed the general perspective of healthcare professionals from all 17 Spanish regions or Autonomous Communities. Each Autonomous Community has its own regional parliament and government, with independent health politics and general state-wide health strategies.

To compare the professionals ‘perception with the available existing resources, between June and July 2012, the on-line questionnaire was also completed by 19 commissioners and managers responsible of the national and regional drug plans (17 commissioners from the regions and 2 commissioners from the autonomous cities Ceuta and Melilla). Commissioners are member boards (heads of addiction services), responsible to promote action policies in the addiction fields in the different Spanish autonomous communities or regions.

### Statistical analysis

In the statistical analysis, frequency tables and percentages were obtained for categorical variables while measures of central tendency and dispersion were calculated for continuous variables (mean, Standard Deviation (SD), minimum and maximum and 95% confidence intervals).

## Results

### Sample characteristics

A total of 659 healthcare professionals working throughout Spain, were recruited to participate in the study. The sample included 55% (n = 286) women and 45% (n = 232) men from 553 centers in 235 cities across Spain. 95.9% of centers provided 1 or 2 participants, 2.6% provided 3 participants, 0.7% provided 4 participants, while only a 0.04% provided 7 participants.

The majority of the sample was of Spanish origin (n = 625, 94.8%). The rest of study participants were Latin American (n = 22; 3.3%), from other European countries (n = 8; 1.2%) and a small proportion (n =4; 0.6%) of other origins. Most of the participants were psychologists (43.40%) or psychiatrists (32.93%), followed by general practitioners (14.57%) and physicians with other specialist degrees (7.59%), whilst a small percentage of professionals had two or more specialties.

In general, diagnosis of dual pathology, performed by psychiatrists, was established according to Diagnostic and Statistical Manual of Mental Disorders, Fourth Edition, Text Revision (DSM-IV-TR)-based.

Regarding network affiliations, 40.5% of participants belonged to the mental health network and 35.4% to the addictions network, while a lower percentage of participants belong to the unified network (mental health and addictions) (13.5%) or to both separated networks (10.3%). Only 0.3% participants were not attached to any network.

From the 19 asked commissioners, a total of 16 (84.2%) answered the questionnaire.

### Knowledge on networks

**Figure 1:**
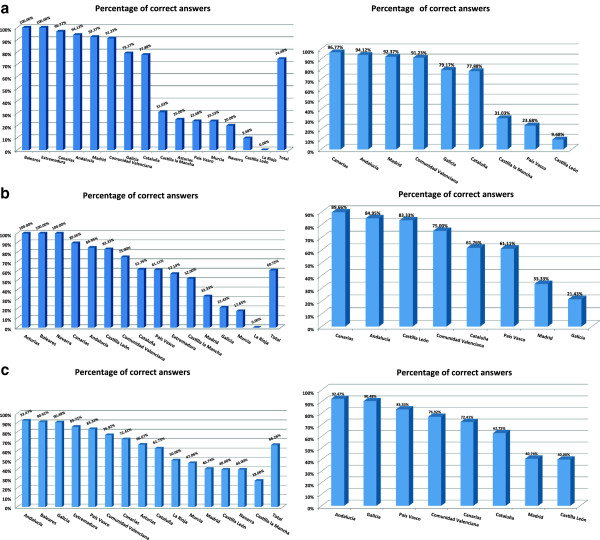
**Degree of professionals’ knowledge on the existing available resources of mental health and addictions networks. (a)**, outpatients programs **(b)** and specific hospitalization units **(c)** for patients with dual disorders.

Regarding the level of knowledge on the mental health and addictions networks (existence of separated networks and/or a unified treatment network), a high degree of agreement between professionals’ perceptions and commissioners’ data (about 75%) was observed (Figure 1a).

**Figure 2:**
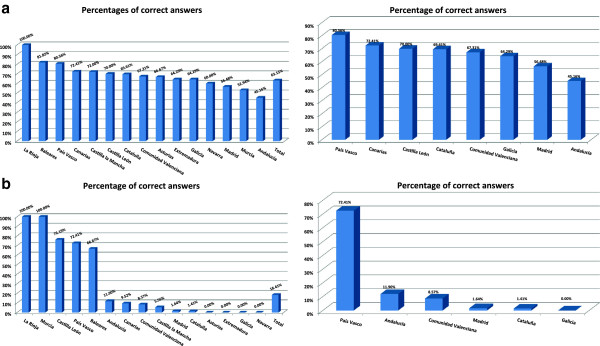
**Knowledge on the available existing detoxification units accepting patients with dual disorder. a)** Degree of knowledge on the existence of detoxification units accepting patients with dual disorder, **b)** Degree of knowledge on the number of detoxification units accepting patients with dual disorder.

**Figure 3:**
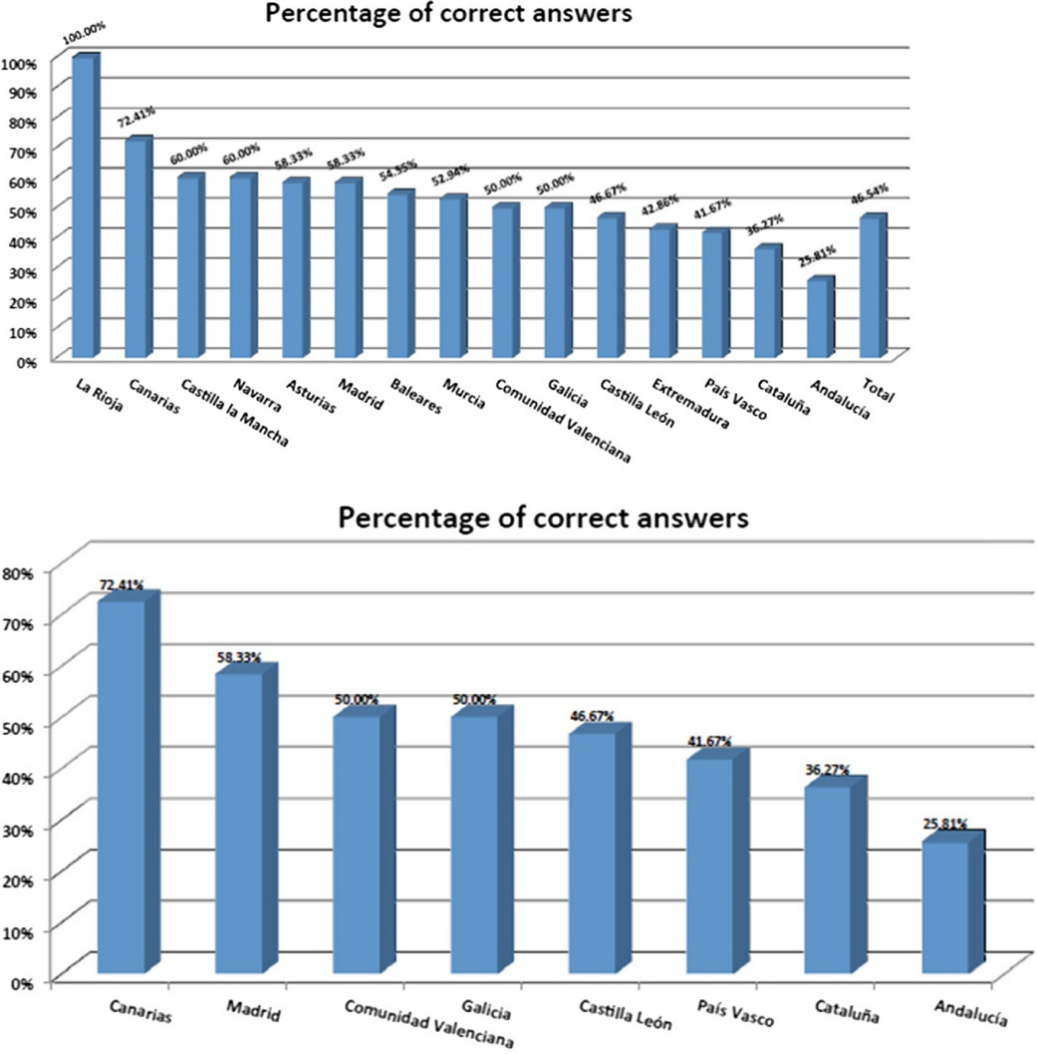
**Knowledge on the available existing psychiatric acute admission units for patients with dual disorder.**

**Figure 4:**
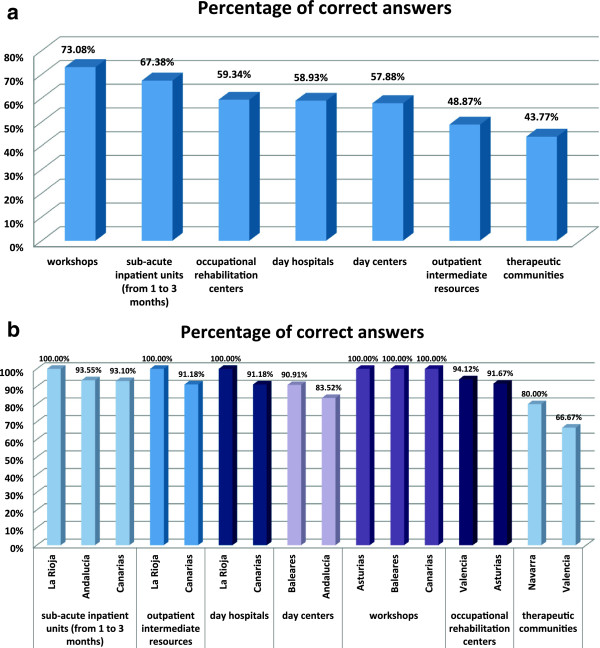
**Professionals’ perceptions on the number of resources for patients with dual pathology. a)** Percentages of surveyed healthcare professionals informed on the number of different resources for dual pathology in their own autonomous community, **b)** Communities with the highest percentage of informed professionals about the number of resources for dual pathology.

**Figure 5:**
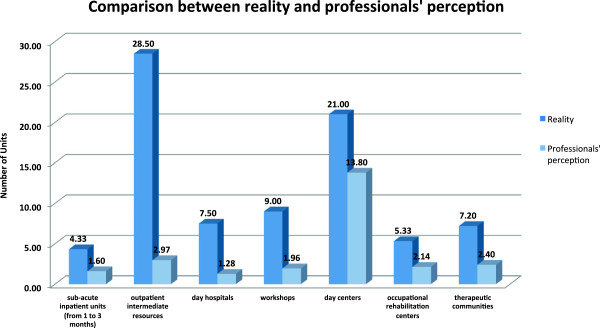
**Comparison between existing resources and professionals’ perception on the number of dual pathology units.** Resources include sub-acute inpatient units, outpatient intermediate resources, day hospitals, day centers, workshops, occupational rehabilitation centers, therapeutic communities.

Knowledge on existing resources for co-occurring disorders.A lower percentage of concordance (about 61%) was found between professionals’ perception and existing resources regarding the available outpatient programs for co-occurring disorders (Figure 1b). In addition, 66.26% of participating professionals answered in agreement with the commissioners’ reports on specific hospitalization units for co-occurring disorders (general adult psychiatry inpatient units intended to offer short-term hospitalization to treat, control or stabilize the individual patient) (Figure 1c).A total of 63.15% of participating healthcare professionals provided accurate answers on the existing detoxification units accepting dually-diagnosed patients (facilities designed to offer medical evaluation and to provide medically-assisted withdrawal treatment to adult patients with dual pathology who may require pharmacological treatment to manage withdrawal symptoms from alcohol or other drugs). In contrast, only 18.41% of professionals correctly indicated the number of these units (Figure 2).Only 46.54% of professionals were correctly informed on the existing psychiatric acute admission units for co-occurring disorders, whilst only 2.54% of these knew the correct number of this type of units available (Figure 3).Study participants were also asked on the number of individual resources in each community for patients with co-occurring disorders. A total of 67.38% of surveyed healthcare professionals were aware of the existence of sub-acute inpatient units (1–3 months of inpatient stay, provided to patients with a co-occurring disorder experiencing an exacerbation or a relapse of their condition), 48.87% of outpatient intermediate resources, 58.93% of day hospitals, 57.88% of day centers, 73.08% of workshops, 59.34% of occupational rehabilitation centers and 43.77% of therapeutic communities accepting patients with dual disorders (Figure 4).For all the different resources, in general, professionals underestimated the number of resources present in their respective communities. In particular, we found a relevant difference between the number of units offered in each community and the number believed to be available by the professionals: 4.33 vs 1.6 for sub-acute inpatient units, 28.5 vs 2.97 for outpatient intermediate resources, 7.5 vs 1.28 for day hospitals, 9 vs 1.96 for workshops, 21 vs 13.8 for day centers, 5.33 vs 2.14 for occupational rehabilitation centers and 7.2 vs 2.4 for therapeutic communities (Figure 5). Finally, we found relevant differences between the Spanish regions in all the questions.

## Discussion

### Importance of findings

In health-services planning and in the evaluation of healthcare needs for the provision of adequate treatment resources, opinions of healthcare professionals are generally sought by healthcare authorities, as a strategy that is receiving increasing attention in recent years [27]. However, the number of studies evaluating the perceptions of professionals on healthcare resources around the world, unfortunately, is still low.

To our knowledge, the present observational study is the first report comparing the perceptions of professionals with data on the existing available integrated resources for the management of patients with co-occurring disorders, since current data is evidencing the need of integrating services for the optimal treatment of co-morbid disorders [17, 28, 29]. Indeed, integrated services for dually diagnosed patients pathology face continuous challenges to find more efficient and effective strategies for improving the overall poor prognosis and outcomes and the rates of morbidity and mortality associated with this condition [8, 10, 13, 16, 21, 28, 30, 31].

Previously, we reported that in Spain specific healthcare resources for the management of co-occurring disorders were clearly insufficient according to the professionals’ perceptions [13, 24]. In view of these results, we compared the perceptions of professionals with data coming from commissioners and managers responsible for the national and regional healthcare plans on drug abuse. Results obtained have evidenced the important gap between reality and professionals’ perceptions on the existence of specific resources for dually diagnosed patients, with systematic underestimation of the number of the existing resources. We also noted important differences among the different Spanish regions, thus highlighting the existing diversity of professionals’ knowledge on specific resources, which could be derived from the independent health politics and general state-wide health strategies of the regions. We think that the implementation of a single national register on this type of resources could avoid this reported lack of information and that information and dissemination campaigns are required to make known the available specific resources for co-occurring disorders.

Although we could not perform comparisons with any other international study, we consider these results of great relevance, since they evidence that, as a consequence of the lack of adequate knowledge by healthcare professionals, currently the available integrated resources are probably underused, thus failing to provide an optimal management of patients with co-occurring disorders. Although the causes of this lack of knowledge have not been addressed, we postulate that it can be at least partly explained by the lack of diffusion or a poor information flow regarding the available resources in each region.

As in other health problems that require an integrated care approach, professionals need to be aware of both local and regional network resources and specific treatment facilities available from each individual institution [32, 33]. Devising a register of available integrated resources for health professionals could be a feasible option to address this issue. As proposed elsewhere, classification of local and community resources based on the treatment offered, professionals in charge, etc. could help professionals working with dually diagnosed patients to identify a quick and targeted access to available resources, according to patient needs [32, 33].

### Limitations and strengths of the study

In the absence of the necessary information, in the present study it is possible that some professionals could have provided incorrect answers due to having misleading information or no information at all. We believe that a register, with a correct classification of resources (integrated/non integrated), would help to revert this tendency. Among the strengths of the study, it is important to consider that this report, based on a large sample of healthcare professionals, gives for the first time, new insights into the real knowledge of professionals attending patients with co-occurring disorders on integrated resources. In fact, being known the complexity of management of these patients, assessing perceptions and knowledge of professionals is of paramount importance for the specific management of the disorder, since these professionals are responsible to select treatments, interventions and resources use [24, 26].

Importantly, we consider that the surveyed sample of Spanish professionals working with co-occurring disorders patients could represent between 5–8% of all the professionals working in the Spanish mental health or addition networks. Of note, the official data corresponding to the existing resources were obtained from the different commissioners, which are the maximum autonomic authorities in this issue.

Among the limitations of this report, we consider that it is possible that professionals participating in this study could be most interested in co-occurring disorders than the others who did not participate, thus their answers could not be representative of the total of professionals. The high percentage of respondent professionals, however, allows us to have a broad picture of the current situation, useful for treatment planning and developing adequate health policies. Further studies should assess the possible causes of the reported differences and the implementation of new strategies, such as a national register of integrated resources for co-occurring disorders.

## Conclusions

Based on the results of the present study, we can conclude that relevant differences exist between the perceptions of professional and existing available resources for co-occurring disorders patients in Spain, thus supporting the need of additional efforts and strategies to register and inform about the existing resources for dually-diagnosed patients. We propose the implementation of a single national register and information and dissemination campaigns to make known the available specific resources for co-occurring disorders.
